# Adapting the Baseline Resilience Indicators for Communities (BRIC) Framework for England: Development of a Community Resilience Index

**DOI:** 10.3390/ijerph21081012

**Published:** 2024-08-01

**Authors:** Christine Camacho, Roger T. Webb, Peter Bower, Luke Munford

**Affiliations:** 1 Division of Population Health, Health Services Research and Primary Care, University of Manchester, Oxford Road, Manchester M13 9PL, UK; peter.bower@manchester.ac.uk (P.B.); luke.munford@manchester.ac.uk (L.M.); 2 Division of Psychology and Mental Health, University of Manchester, Oxford Road, Manchester M13 9PL, UK; roger.webb@manchester.ac.uk

**Keywords:** community resilience, measurement, composite index

## Abstract

Community resilience results from complex interactions between people, places, and environments. Measuring community resilience aligns with policy objectives to enhance resilience to adverse events and address spatial disparities. The Baseline Resilience Indicators for Communities (BRIC) is a composite index used to measure resilience. This study adapts the BRIC approach to develop a Community Resilience Index (CRI) for England. A systematic review informed indicator selection, and principal components analysis was used to define sub-indices and allocate weightings. The resulting CRI comprised 44 indicators across five domains, quantifying the resilience of English local authorities. Geographical comparisons were made using *t*-tests and ANOVA, and the CRI was validated against the Index of Multiple Deprivation (IMD). The mean CRI score for local authorities in England was 83.1, ranging from 53.3 to 108.9. Resilience scores showed spatial patterning, with London and the South East scoring highest. The North had lower CRI scores than the Midlands and South (*p* = 0.022). Coastal and rural areas also showed lower resilience (*p* < 0.001). CRI and IMD were inversely correlated (r = −0.564, *p* < 0.0001). This study contributes to the literature by providing the first detailed assessment of community resilience in England using an adapted BRIC framework. The CRI provides a framework for measuring community resilience in England and could be used to explore associations with health outcomes and guide funding allocation.

## 1. Introduction

Community resilience arises from complex interactions between the people in a place, the economic assets at their disposal, and the supporting institutions and infrastructure. Definitions abound, but a tripartite view is emerging in the literature, in which community resilience is hypothesised to mitigate the impacts of shocks, accelerate recovery, and reduce the negative impact of future adverse events [1].

The concept of building community resilience has risen to prominence largely as a policy response to the impact of natural hazards [2]. However, community resilience is closely aligned with wider public health policy on place-based and community-centred approaches [3]. These activities are central to building community resilience and are also recognised as a means of reducing health inequalities [4]. Similarly, policy on place-based approaches recognises the multi-factorial causes of health inequalities and the importance of addressing these at a population rather than individual level [5]. In this paper, community resilience is defined as the capabilities required to enhance the ongoing vitality of communities [6] and their ability to cope, adapt, and transform in response to crises.

Although the concept of resilience has been applied in many fields, there is an ongoing debate regarding the limitations and potential drawbacks of resilience as a framework for understanding and addressing the needs of communities. These issues have been extensively discussed in the literature [7,8,9], so only the key critical perspectives relevant to this study are outlined here. Firstly, it is important to acknowledge that resilience does not exist in a political vacuum. One critique is that a focus on building community resilience is influenced by neoliberal ideologies, placing an onus on local communities to cope and adapt in the face of reducing support from the state [10]. This shift in responsibility could lead to a lack of infrastructure support and national economic policies required to build resilience. Secondly, while we propose the CRI as a measure of baseline resilience, resilience is increasingly conceptualised as a process. Resilience in communities therefore arises through a bidirectional relationship between individuals and their environments [11], and importantly, it will be influenced by past shocks and the scars that these have left on communities. Finally, a common critique is that resilience is often ambiguously defined, leading to inconsistencies in its application and measurement [12]. These perspectives provide a critical backdrop against which the CRI is developed and applied in this study.

Measurement of community resilience is an emerging area of research, largely driven by policy interest and the need to assess interventions aimed at increasing community resilience. The measurement of resilience poses many challenges. It is a phenomenon that cannot be directly observed and is inferred based on constructs such as exposure to risk and positive adaptation [13]. The field of community resilience measurement has developed in recent years, with composite indices, which combine multiple indicators into a single summary measure, emerging as a popular methodology.

In 2010, Cutter et al., developed the Baseline Resilience in Communities (BRIC) framework. The BRIC model is now the most replicated and mature approach for measuring community resilience [14,15] and has been deployed by the US Federal Emergency Management Agency [16]. The BRIC framework has been adapted for use in at least 15 countries outside of the USA where it was originally developed, but it has not been applied in the UK [17].

The UK consists of England, Northern Ireland, Scotland, and Wales, with 84% of the UK’s population residing in England. The UK is not particularly prone to natural hazards; however, the growing threat of climate change is likely to bring more extreme weather events to all parts of the globe. Currently, the UK faces a number of chronic shocks that have had a substantial impact on the UK, including the global financial crisis (2008), the social and economic impacts of leaving the European Union (2016), the COVID-19 pandemic (2020), and an ongoing cost of living crisis (2021) [18]. Developing community resilience is a key UK government objective in addressing both acute and chronic shocks [2,19]. The concept of community resilience is closely aligned with the ‘capitals’ outlined in the UK Government’s Levelling Up White Paper: physical, human, intangible, financial, social, and institutional [20]. The paper describes these capitals as the drivers of spatial disparity and sets out an aspiration for all communities in the UK to be richly endowed with all these capitals.

The aim of this paper is to adapt the BRIC model to develop a composite Community Resilience Index (CRI) for England. The CRI is focused primarily on chronic rather than acute shocks. This decision is grounded in the context of the UK and our definition of community resilience. By focusing on chronic shocks, the CRI aims to provide a comprehensive framework for assessing and enhancing communities’ abilities to navigate and thrive in the face of prolonged challenges. This public health approach to community resilience recognises the importance of addressing the underlying structural and systemic inequalities that contribute to chronic shocks and the need to build resilience that extends beyond immediate crises to encompass the long-term well-being and sustainability of communities in the UK.

## 2. Materials and Methods

The Organisation for Economic Co-operation and Development (OECD) composite indicator checklist is a well-recognised framework that outlines ten steps for composite index construction [21] (Table 1). This framework was used as a methodological guide to construct the CRI for England. The methods section is organised according to these ten steps. Data were analysed using Stata version 17 [22].

### 2.1. Theoretical Framework and Definition of Community

This subsection introduces the theoretical framework that underpins the study and defines the concept of community as used in this research. The Disaster Resilience of Place (DROP) model is the theoretical basis for the BRIC framework [23]. The DROP model was designed to evaluate resilience in the context of natural hazards, but the authors state it is also applicable to chronic shocks. The BRIC operationalises the ‘antecedent conditions’ element of the DROP framework, which describes the inherent resilience conditions of a place before a ‘shock’ occurs. The DROP model is cyclical and depicts how the antecedent conditions of a place interact with a shock to produce an impact. A more resilient community will be able to implement better coping responses to mitigate the impact of the event.

A key assumption of the DROP model is that the social resilience of a place is determined by the interconnection of natural systems, social systems, and the built environment. Communities are described in the DROP model as “the totality of social system interactions within a defined geographic space such as a neighbourhood, census tract, city, or county” [23]. Local authority districts (LADs), which are similar in population size to US counties, are used to define communities in this study [24].

England comprises nine regions, within which sit 309 LADs, with a median population size of 142,000 (IQR 103,900–225,300) in 2021. London is defined as the 32 boroughs that comprise the Greater London conurbation. The City of London (the financial district, with a population of 8600 residents) and Isles of Scilly (with a population of 2100 residents) were excluded from our study due to their very small population sizes. The resulting dataset contained 307 LADs.

### 2.2. Indicator Selection

The six dimensions from the DROP model (social resilience, economic resilience, institutional resilience, infrastructure resilience, community capital, and environmental resilience) were used as an initial framework for the selection of variables. A thematic analysis of 1024 indicators from a systematic review and other relevant studies and composite indices was carried out to inform indicator selection (Table S1, Supplementary Materials) [17,23,25,26,27,28,29]. Indicators were chosen to give optimal coverage of the identified themes (Table 2). Additionally, the following principles were used to guide indicator selection: applicability to the English context, availability of data at local authority level, and relevance to longer-term adversity rather than specifically to acute hazards. Indicators that were measuring health outcomes in the population were purposefully excluded, as health is hypothesised to be an outcome of community resilience [26]. Finally, since principal component analysis (PCA) was used to construct the index, an empirical approach was used to select the appropriate number of indicators. PCA guidelines suggest having 5 to 10 observations per variable [30]. With a sample size of 307 LADs, the acceptable range for the CRI is 31 to 61 indicators. The CRI comprises 44 indicators, which are described in Table 3.

The themes and indicators identified in our study align with and expand upon those applied in foundational research for this topic. For example, the original BRIC study examined 36 variables across five sub-domains [25]. Subsequent studies have varied significantly in the number and type of indicators used, ranging from 3 to 57 indicators and covering between 1 and 6 sub-domains. This variability reflects both methodological quality and the diverse contexts and specific resilience needs addressed in different regions. Our thematic analysis incorporated indicators from a wide range of studies, including those conducted in Pakistan, Germany, Zimbabwe, China, and the USA, among others [17]. The inclusion of themes such as “Demographics” and “Civic engagement” can be traced back to the candidate indicators proposed in the DROP framework [23]. However, other themes like “Food” and “Culture/sports” have emerged from subsequent interpretations of this framework. For instance, the BRIC adaptation for Norway included the following two indicators we themed as culture/sports: cinemas, youth centres, and clubs per thousand people; and museums, libraries, zoos, and botanical gardens per thousand people. Similarly, a BRIC study in Zimbabwe included seven indicators relating to food across the social and economic domains [31].

**Table 3:** Indicators included in the Community Resilience Index for England.

	Theme	Indicator Description	Justification	Source	Direction
	**Social**				
1	Demographic	Living alone—Percentage of households occupied by one person (2021)	Morrow (2008) [32]	ONS [33]	Low is good
2	Demographic	Age—Dependency ratio—Population aged 0–16 and 65+/population aged 17–64 (2021)	Morrow (2008) [32]	ONS [34]	Low is good
3	Demographic	Population density—number of usual residents per square kilometre (2021)	UK Government (2021) [28]	ONS [35]	High is good
4	Demographic	Inward migration—Percentage of the population who are inward migrants (domestic or international) in the last 12 months (2021)	Cutter et al. (2010) [25] Morrow (2008) [32]	ONS [34]	High is good
5	Equity	IMD gap—Population weighted mean difference in Index of Multiple Deprivation score between LSOAs in each LA (2020)	Norris et al. (2008) [36]	OHID [37]	Low is good
6	Education/ skills	Adult skills—Qualification index score is the average value of individuals aged 16 based on their highest level of qualification (2021).	Norris et al. (2008) [36]	ONS [38]	High is good
7	Language/ communication	Language—Speaks English as a first language or very well (2021)	Cutter et al. (2010) [25] Morrow (2008) [32]	ONS [39]	High is good
8	Language/ communication	Digital propensity index—Populated weighted average of online-first census data returns	Tierney (2009) [40]	ONS [41]	High is good
9	Transport	Car availability—Percentage of households with no access to a car or van (2021)	Cutter et al. (2010) [25] Tierney (2009) [40]	ONS [42]	Low is good
10	Food	Food insecurity—Modelled percentage of households experiencing hunger or struggling to access food (2021)	Cutter et al. (2014) [43]	Moretti et al. [44]	Low is good
	**Economic**				
11	Labour market	Employment—Percentage of population aged 16–64 in employment (2021)	Cutter et al. (2010) [25] UK Government (2021) [28]	ONS [45]	High is good
12	Labour market	Economic inactivity—Percentage of the population aged 16–64 who are economically active (2021)	Cutter et al. (2010) [25]	ONS [45]	Low is good
13	Labour market	Employment—Distribution of employment across sectors (2021)	Cutter et al. (2010) [25]	ONS [46]	High is good
14	Equity	Gini index—Inequality in household income within LADs (2020)	Cutter et al. (2010) [25] Norris et al. (2008) [36]	ONS [47]	Low is good
15	Equity	Gender pay gap—Mean difference between average hourly earnings between men and women (2020–2022)	Cutter et al. (2014) [43]	ONS [48]	Low is good
16	Income/ savings /debt	Income—Gross Disposable Household Income (GDHI) (2021)	Aldrich and Meyer (2015) [49]	ONS [50]	High is good
17	Income/ savings /debt	Percentage of children in absolute low-income families (2021/22)	Morrow (2008) [32]	OHID [51]	Low is good
18	Income/ savings /debt	Fuel poverty—Modelled estimates of the proportion of households in fuel poverty, 2020	Morrow (2008) [32]	Department for Business, Energy and Industrial Strategy [52]	Low is good
19	Commerce/ retail	Loans and overdrafts to small- and medium-sized enterprises (SME) per capita (2021)	Norris et al. (2008) [36]	UK Finance [53]	High is good
20	Commerce/ retail	High street vibrancy—difference in percentage of vacant retail units (2019 vs. 2023)	Hill (2023) [54]	CDRC [55]	Low is good
21	Housing	Housing affordability—Ratio of lower quartile house price to lower quartile gross annual workplace-based earnings (2021)	Norris et al. (2008) [36]	ONS [56]	Low is good
22	Economic output	Productivity—Nominal smoothed Gross Value Added (GVA) per hour worked (2021)	UK Government (2021) [28]	ONS [57]	High is good
	**Institutional**				
23	Local government	Municipal spending—Public health grant allocation per head of population (2021/22)	Plough et al. (2013) [58]	MHCLG [59]	High is good
24	Local government	Total core spending power/dwelling (2021/22)	Platts-Fowler and Robinson (2016) [60]	DLUHC [61]	High is good
25	Local government	Non-ringfenced reserves as percentage of service spend (2022/23)	Platts-Fowler and Robinson (2016) [60]	DLUHC [61]	High is good
26	Civic engagement	Lottery—Big Lottery community funding per head of population (2021–2023)	Aldrich and Meyer (2015) [49]	The National Lottery Community Fund [62]	High is good
27	Education/skills	Pupil absences—Percentage of school sessions missed due to pupil absence (2021/22)	Ellis et at (2022) [63]	OHID [51]	Low is good
28	Education/ skills	School quality—Percentage rated good/outstanding of those inspected	Ellis et at (2022) [63]	Department for Education [64]	High is good
	**Infrastructure**				
29	Housing	Household overcrowding—Percentage of number with an occupancy rating of less than −1 (2020)	Ellis et at (2022) [63]	Health index [65]	Low is good
30	Housing	Households in temporary accommodation (2020–2021)	Ellis et at (2022) [63]	MHCLG and DLUHC [66]	Low is good
31	Transport	Average minimum travel time to key services by public transport (2019)	Tierney (2009) [40]	Department for transport	Low is good
32	Transport	Distance to sports and leisure facilities	Tierney (2009) [40]	Health index [65]	Low is good
33	Language/ communication	Broadband—Percentage of premises that do not have access to services above 10 Mbit/s from fixed broadband (2020)	Tierney (2009) [40]	Health index [65]	Low is good
	**Environmental**				
34	Green space/forests	Private outdoor space—Percentage of addresses (houses and flats) with private outdoor space (2020)	Pamukcu-Albers et al. (2021) [67]	Health index [65]	High is good
35	Green space/forests	Access to green space—Average distance to nearest park, public garden, or playing field (m) (2020)	Pamukcu-Albers et al. (2021) [67]	ONS [68]	Low is good
36	Waste/ pollution	Air pollution—Territorial carbon dioxide (CO_2_) emissions estimate within the scope of influence of Local Authorities 2020 (kt CO_2_e)	Fazey et al. (2018) [69]	Department for Business, Energy and Industrial Strategy [70]	Low is good
	**Community Capital**				
37	Demographic	Population churn—Proportion of households with change in occupiers (2016–2021)	Cutter et al. (2010) [25] Norris et al. (2008) [36]	CDRC [71]	Low is good
38	Civic engagement	Charities—Registered charities per 100,000 population (2022)	Cutter et al. (2010) [25] Aldrich and Meyer (2015) [49]	Charity Commission [72]	High is good
39	Civic engagement	Assets of community value—Registered assets of community value/100,000 population	Norris et al. (2008) [36]	Plunkett UK [73]	High is good
40	Political engagement	Electoral turnout—Percentage of population who voted in the 2019 general election	Cutter et al. (2010) [25] Morrow (2008) [32]	House of Commons Library [74]	High is good
41	Religion	Religious affiliation—Percentage of population who are religious adherents (2021)	Cutter et al. (2010) [25] Aldrich and Meyer (2015) [49]	ONS [75]	High is good
42	Well-being	Life satisfaction—Average score of survey respondents (aged 16 years and over) when asked how happy they felt on the previous day (2020)	Norris et al. (2008) [36]	Health index [65]	High is good
43	Culture/sports	Municipal spending—Budget allocation for cultural and related services per head of population (2021/22)	Vårheim (2017) [76]	Department for Levelling Up, Housing, and Communities [77]	High is good
44	Antisocial behaviour	Crude rate of noise complaints per 1000 population (2020)	Dzhambov et al. (2017) [78]	Health index [65]	Low is good

### 2.3. Imputation of Missing Data

For most of the indicators (*n* = 33, 75%), datasets were complete at the local authority district level. For a further 7 indicators (food insecurity, lottery funding, households in temporary accommodation, access to green space, Gini index, SME loans, and electoral runout), complete data were available but required processing to map these to 2022 LAD boundaries. For 2 indicators (registered charities and public health grants), data were only available at the upper-tier local authority level; for these indicators, LAD values were imputed using population-weighted means. For the core reserves indicator, data for 2020/21 were missing for 3 LADs (Bromsgrove, Corby, and Redditch); these values were replaced by data from the previous year. Finally, in the high street vibrancy dataset, data from 2019 were missing for 2 LADs (Forest of Dean and South Staffordshire). These values were imputed by calculating the mean baseline value from the CIPFA Nearest Neighbour groupings for these two LADs [79].

### 2.4. Multivariate Analysis

PCA with varimax rotation was applied to allocate indicators to sub-indices and inform the weighting scheme. Meaningful results from PCA depend on the interrelatedness of variables in the dataset. If the correlations between variables are minimal, the likelihood of sharing common components diminishes. Kaiser–Meyer–Olkin Measure (KMO) of Sampling Adequacy and Bartlett’s test of sphericity were used to evaluate indicator correlations, gauging the dataset’s suitability for PCA. Parallel analysis was applied to the PCA results to decide how many components should be retained. This method ensures that the retained components are empirically justified, reflecting the underlying structure of the data rather than a predetermined number of domains.

### 2.5. Normalisation and Transformation

Variables were standardised prior to PCA and subsequently transformed using percentile ranks before index construction. The ranking was reversed for indicators where a lower score is better, so that a higher rank is associated with higher community resilience for all indicators.

### 2.6. Aggregation and Weighting

Indicators were categorised into sub-indices based on the principal components to which they exhibited the highest loading. Within each sub-index, indicators were weighted according to their normalised squared loading for the associated factor. Sub-index scores were a weighted mean of the indicators comprising it. Sub-indices were weighted by multiplying them by the eigenvalue for the respective PCA component. The CRI was generated by summing the weighted sub-indices (Equation (1)). PCA is a well-established method for weighting indices, and this specific methodology has been used in the construction of other composite indices [80,81].

(1) $${SubIndex}_{i}=\sum_{j=1}^{n_{i}}\frac{X_{ij}\times W_{ij}}{n_{i}}$$

where X_ij_ represents each indicator within sub-index_i_; W_ij_ is the weight assigned to each indicator within sub-index_i_; and j is the index for the indicators within sub-index_i_, ranging from 1 to n_i_.

$$CRI=\sum_{i=1}^{M}{Subindex}_{i}\times W_{i}$$

where CRI is the weighted composite index; M is the total number of sub-indices; W_i_ is the weight assigned to sub-index; and i is the index for the sub-indices, ranging from 1 to M.

### 2.7. Sensitivity Analysis

As a sensitivity analysis, the CRI was reconstructed using the indicator grouping and weighting methodology outlined in the original BRIC paper [25]. Following normalisation and transformation (as described above), the sub-indices were formed using indicators from the original six dimensions. The sub-indices were combined into a composite index without applying any weightings (Equation (2)). The CRI scores from the unweighted ‘BRIC’ model were compared with the PCA-weighted index using pairwise correlation analysis.

(2) $${SubIndex}_{i}=\sum_{j=1}^{n_{i}}\frac{X_{ij}}{n_{i}}$$

where X_ij_ represents each indicator within sub-index_i_ and j is the index for the indicators within sub-index_i_, ranging from 1 to n_i_.

$$CRI(BRIC)=\sum_{i=1}^{M}{Subindex}_{i}$$

where CRI is the weighted composite index; M is the total number of sub-indices; and i is the index for the sub-indices, ranging from 1 to M.

### 2.8. Back to the Data

The relative weighting of the index was assessed by calculating the mean contribution across all LADs of each sub-index to the overall CRI. The resulting percentage values represent the average contribution of each sub-index. This served as a check on the weighting scheme to ensure that the sub-indices were differentially represented in the CRI as intended.

Previous research has identified that people living in northern and coastal areas of England experience poorer outcomes across a range of measures [82,83,84]. Differences in community resilience have been identified between urban and rural areas [85]. To understand the variation in outcomes, comparisons were made between different regions and classifications within England. Specifically, outcomes were compared between the North of England and the rest of England, coastal versus inland areas, and urban versus rural LADs.

The North of England was defined to include the North East, North West, and Yorkshire and The Humber regions, with a combined population of 15.5 million. A LAD was classified as ‘coastal’ if more than half of its population resided in coastal lower super output areas (LSOAs), as defined in the Chief Medical Officer’s report on coastal communities [86]. LADs were classified as being predominantly urban, urban with significant rural areas, or predominantly rural in accordance with existing national statistics [87]. These comparisons were conducted using *t*-tests and ANOVA.

### 2.9. Validation

The CRI scores and sub-indices were compared with the Index of Multiple Deprivation (IMD), which is commonly used in England as an area-level measure of deprivation. The IMD is a composite indicator comprised of the following seven domains: income, employment, education, health, crime, barriers to housing and services, and living environment [88]. Summary IMD domain scores for LAs were constructed using population-weighted scores from LSOA-level data; scores were then transformed to ranks [89]. A higher rank indicates a higher level of deprivation. Correlation analysis was used to compare ranked CRI scores and sub-indices with IMD. Since deprivation and resilience are opposing but not opposite concepts, a moderate negative correlation between IMD and CRI was anticipated. A linear regression model was used to generate predicted CRI scores based on IMD rank. Actual and predicted CRI scores were compared to identify LADs with positive and negative outlying CRI scores based on deprivation.

### 2.10. Visualisation

**Figure 1:**
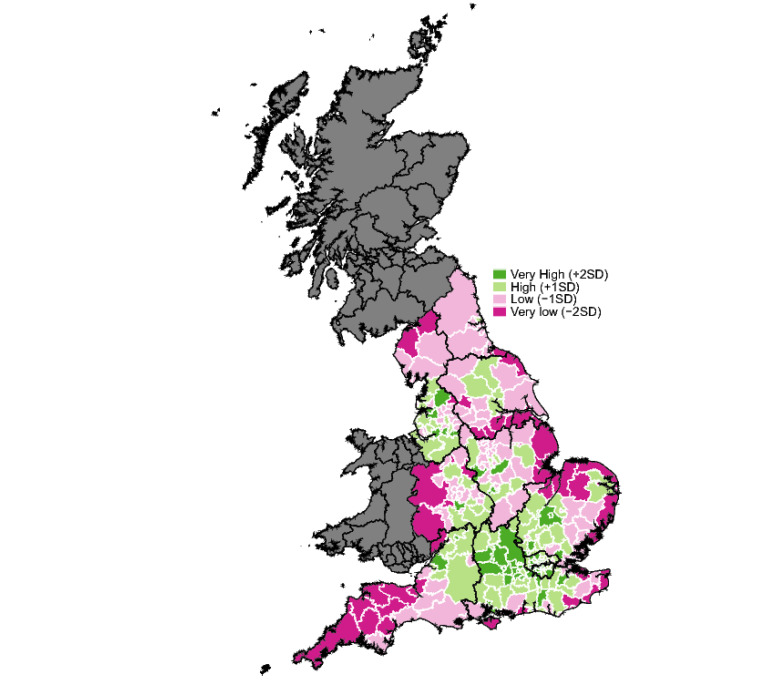

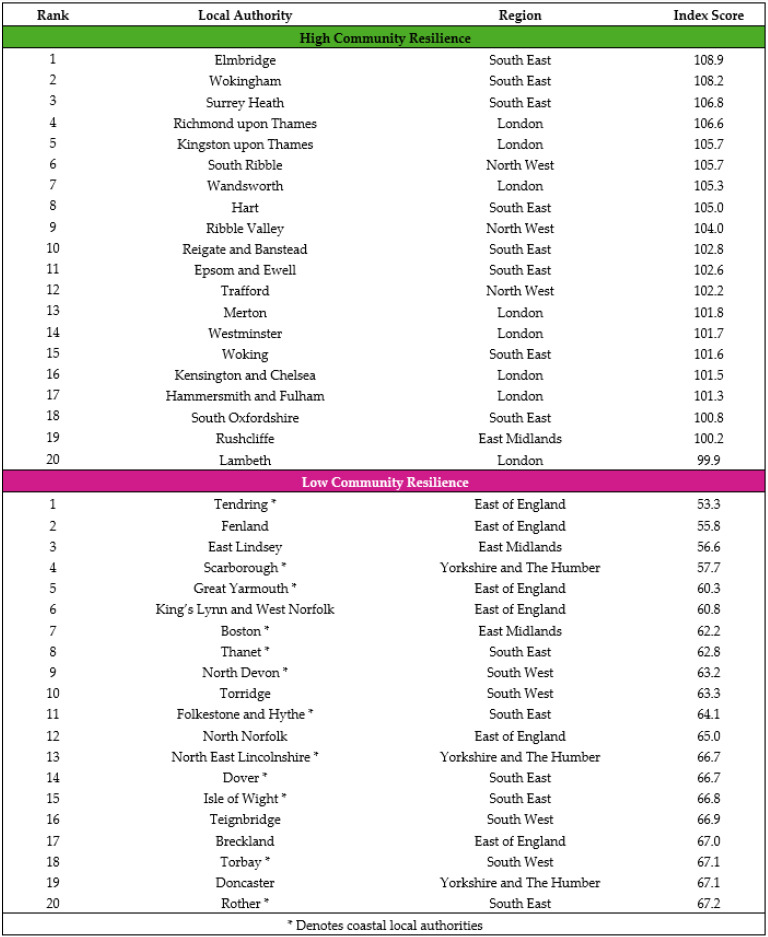
Map showing composite resilience results in England, with scores for the top and bottom 20 local authority districts.

The CRI and the sub-indices were mapped using LAD shapefiles (2022) [24]. Data were plotted as standard deviations from the mean value. To provide clarity on the coastal boundaries of England, the CRI map (Figure 1) also includes Scotland and Wales.

## 3. Results

The Bartlett (*p* < 0.0001) and KMO (0.88) tests indicated that the data are suitable for PCA. Parallel analysis indicated that five components should be retained from the indicator set (adjusted eigenvalues > 1). Variables were allocated to the component in which they had the greatest loading. The five sub-indices resulting from PCA comprised variables spanning multiple domains, resulting in inter-dimensional facets of community resilience. This reduction from the initial six BRIC domains to five sub-indices was based on the empirical evidence provided by PCA and parallel analysis, ensuring that the retained components accurately reflect the underlying data structure. Each sub-index has been named based on its constituent indicators (Table 4). As intended, the weighting scheme resulted in the sub-indices contributing differently to the composite index. Sub-Index 1 comprised on average 39.6% of the CRI score; the relative contributions for the other sub-indices were 28.1%, 12.0%, 11.5%, and 8.8%, respectively.

### 3.1. Spatial Patterning

The mean CRI score for LADs in England was 83.1 (SD 10.8). Scores ranged from 53.3 (Tendring, East of England) to 108.9 (Elmbridge, South East). Spatial variations were evident in the distribution of CRI scores, with coastal areas featuring heavily in the lowest ranking areas and most of the highest-ranking areas being in London and the South East (Figure 1). At a regional level, there were significant differences between regions (*p* < 0.001). London had the highest score (95.2) and Yorkshire and The Humber the lowest score (75.2) (Table 5). The average CRI scores for the North were lower than the Midlands and South (*p* = 0.022). Coastal LADs had an average CRI score of 76.0, which was significantly lower than inland areas 84.9 (*p* < 0.001). CRI scores showed a gradient across the urban–rural spectrum, with increasing rurality associated with significantly lower resilience (*p* < 0.001). Index and sub-index scores for all LADs are included in Table S2 (Supplementary Materials).

**Figure 2:**
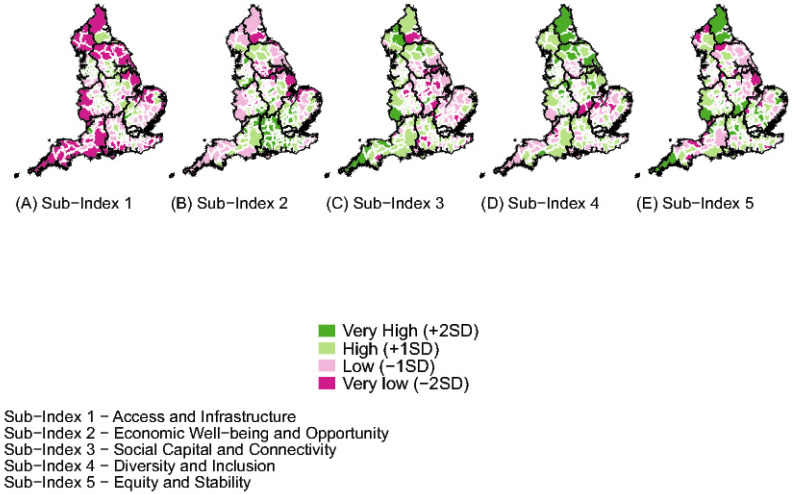
Map showing Community Resilience Sub-Index results in England.

The CRI sub-index scores provide further information on the spatial patterning observed in the composite index score (Figure 2). London achieved the highest score in Sub-Index 1, which assesses access to essential services and infrastructure, followed by the North West and North East. The North of England scored higher in this domain than the rest of the country (*p* = 0.014); conversely, coastal and rural areas scored lower. Sub-Index 2 reflects economic conditions and opportunities, with the South East and London scoring highest, indicating robust economic activity and employment opportunities. The North overall and coastal areas both had lower scores, indicating lower economic resilience.

In Sub-Index 3, which evaluates social capital and connectivity, London again scored highest. Despite regional variations (*p* < 0.001), there was no significant North–South– divide (*p* = 0.16) or difference between coastal and inland areas (*p* = 0.5), although rural areas scored lower, possibly due to poor broadband connectivity and limited access to SME loans. Sub-Index 4 focuses on diversity and inclusion, with higher scores for the North of England (*p* < 0.001) and rural areas (*p* = 0.005), while coastal areas had significantly lower scores (*p* < 0.001). Sub-Index 5 examines equity and stability, with London scoring highest and the North East lowest. Northern and coastal areas scored lower in this domain, but urbanicity did not significantly affect the scores.

### 3.2. Index Validation

Table 6 shows correlation coefficients between IMD and the CRI and its sub-indices. The negative correlation coefficient (−0.564, *p* < 0.0001) between the IMD index and the overall CRI indicates an inverse relationship, as expected, given the definition of deprivation and resilience. Sub-index 2, Economic Well-being and Opportunity, has a strong negative correlation (−0.821) with IMD. The different sub-indices of the CRI show varying correlations with the IMD, suggesting that the CRI adds value by measuring factors related to but not identical with deprivation.

The linear regression model predicting CRI scores from IMD identified positive and negative outliers; the top 10 of these are shown in Table S3 (Supplementary Materials). Positive outliers are LADs where the CRI score was higher than predicted from IMD. For example, Lambeth, located in London, had a CRI score of 99.9 compared to the predicted score of 78.0. Moreover, Fenland in Cambridgeshire was a negative outlier, scoring 55.8 compared to the predicted score of 78.0. Lambeth and Fenland have adjacent IMD ranks, yet there was a large difference in CRI scores. Nine of the top ten positive outliers are situated in London, suggesting there may be a capital city effect where economic, political, and cultural advantages boost community resilience irrespective of deprivation.

### 3.3. Sensitivity Analysis

Spearman’s rank correlation of the PCA-weighted CRI and unweighted CRI score ranks showed a strong positive correlation (r = 0.82, *p* < 0.001). However, the comparison of these scores at a geographical level showed significant differences in the rankings across all measures (Table S4, Supplementary Materials). The mean rank for the North East region was 207.3 (SD 52.2) in the weighted index and 153.9 (SD 78.8) in the unweighted index, meaning the region received lower resilience scores using the weighted methodology.

Evaluating the relative accuracy of these indices presents a challenge, given the absence of an external validation measure. However, in contrast to the BRIC-CRI, the weighted CRI shows significantly higher resilience in the Midlands/South compared to the North. Given the well-documented existence of a North–South divide in many socioeconomic metrics [82,83,84], the weighted index may offer a more valid approach for measuring community resilience.

## 4. Discussion

The CRI provides a framework for assessing community resilience in England across multiple dimensions, offering an empirical measure of the capacity of communities to withstand and recover from adverse events. The CRI scores showed significant spatial patterning, with coastal areas featuring heavily in the lowest-ranked areas and London and the South East comprising many of the highest-ranked LADs. The North of England had lower average CRI scores compared to the Midlands and South. A clear urban–rural gradient was observed, with rural areas having significantly lower resilience. The CRI sub-index scores reveal distinct spatial patterns in access to services, economic conditions, social capital, diversity, and equity across regions. These scores highlight regional disparities in various domains, with London generally scoring more highly than rural and coastal areas. The CRI showed a moderate negative correlation with IMD, indicating that the CRI adds value by providing new information rather than simply serving as an inverse measure of IMD.

The CRI is proposed as a baseline measure of community resilience, but it is crucial to understand that this baseline, as we report, is shaped by historical and ongoing socio-political factors. The resilience scores for communities in England were measured during a period of recovery from the COVID-19 pandemic, amidst a backdrop of government austerity that has detrimentally impacted public services. Further economic shocks such as Brexit, the war in Ukraine, and high government borrowing have exacerbated these challenges, leading to a cost-of-living crisis with rising inflation and slowed growth. Resilience is a dynamic process for which there can never be a ‘true’ baseline. Less resilient communities today may be so due to scars from earlier shocks, such as deindustrialisation, which have eroded their capacity to cope, adapt, and transform in the face of adversity. This historical context is crucial for understanding the current state of community resilience and underscores the need for targeted interventions. Addressing these challenges requires both bottom–up approaches, such as community empowerment, and top–down strategies, including state action to provide the necessary infrastructure and economic opportunities to enable these communities to thrive.

### 4.1. Strengths and Limitations

We believe this is the first published study to measure community resilience in England using a composite index. Composite indices are valuable in measuring complex phenomena such as resilience, but a robust methodology is essential in providing confidence in the resulting index. The main strength of this study is that the CRI was constructed by following all ten steps of the OECD composite indicator guidance [21]. In the original BRIC study, there was evidence of application for five out of ten of the OECD composite indicator guidance steps [25]. A systematic review including 32 studies that used or adapted the BRIC showed variation in the methodological quality of composite indicator development (median 6/10, range 2–9 steps) [17].

One of the key strengths of this study is the data-driven approach to index construction and inclusion of a sensitivity analysis to compare the CRI constructed using data-informed sub-indices and weightings with the original BRIC methodology. Sensitivity analysis and the use of multivariate methods to inform weightings are two of the OECD steps that were inadequately applied in other studies [25]. Although the two resulting indices were highly correlated, the sensitivity analysis revealed significant differences in the composition of sub-indices and spatial patterning of community resilience. In our thematic review of 1024 BRIC indicators, we observed several instances of repetition and inconsistency in the classification of indicators across dimensions. The original BRIC methodology’s subjective nature in assigning indicators to sub-domains potentially introduces inconsistencies. Moreover, this method does not account for the underlying statistical properties of the dataset, which PCA addresses. Consistent with the results of this study, other BRIC studies that report on the construction of a PCA-weighted index as a sensitivity analysis found that the resulting sub-indices were very different in composition to the original BRIC subdomains [43,90]. Our sensitivity analysis goes beyond merely examining the overall correlation between a PCA-style index and the BRIC index, identifying significant differences in CRI outcomes between the two methods.

There are several important limitations to this study. Communities are defined along administrative boundaries, as this enables the use of existing datasets. However, it is important to note that LADs in England, with a median population size of 142,000, may not align with many individuals’ perceptions of what constitutes a community. Two recent studies have attempted to address this issue by downscaling the BRIC to smaller geographical areas [91,92]. While these approaches are promising, the authors reported challenges related to the reconstruction of the BRIC methodology at a sub-county level, including labour-intensive processes, data availability and quality variability, and the need for advanced geospatial analytical skills. While the top–down allows standardised comparisons of multiple areas, it poses challenges in capturing the more subjective aspects of community resilience, such as social capital and a sense of belonging. The indicators included in the community capital domain act as proxies for this, but there is debate as to how effectively social capital can be captured through objective measures [49]. Additionally, we were limited by the availability of reliable data for certain indicators, such as individual debt and crime rates. While we have justified the choice and direction of our indicators, there are nuances to consider. For instance, civic engagement measured by the number of charities could reflect higher need in some areas, and while religious affiliation could offer protective effects for community resilience, its impact may vary in an increasingly secular society.

Community resilience is a multifaceted concept that cannot be directly measured. Composite indices, such as the CRI, are useful tools for approximating complex constructs, but external validation remains a significant challenge. Correlating the CRI with the IMD provides some assurance, as the indices were inversely related as expected, but this does not confirm that the CRI is capturing the true essence of community resilience.

A limitation of adopting a data-driven approach is the reduced scope for participatory input, such as engaging community members, local leaders, and experts with the index development process. This study used a systematic review and thematic analysis of over 1000 indicators to inform the indicator selection process. When the BRIC was adapted for use on the Sunshine Coast, Australia, the indicators closely followed those used in the original BRIC study, with substitute indicators selected where the existing ones were deemed unsuitable [93]. The selection of indicators was undertaken in consultation with representatives from the Sunshine Coast Regional Council and Emergency Management Queensland. An adaptation of the BRIC in Norway used an academic focus group to guide the initial selection of indicators [90].

An empirical approach was used in index weightings for the CRI, informed by the PCA. There are numerous examples of studies using an ‘equal weighting’ assumption [85,90,93,94], for which there is limited theoretical evidence [95]. However, there are also BRIC studies that have used the analytic hierarchy process (AHP), a subjective method for decision-making based on pairwise comparisons, to elicit expert opinion in order to derive weightings for variables [96,97,98]. While our method represents a progression from equal weighting by leveraging empirical data, it is important to note that there is no single agreed-upon method for weighting an index [81]. Participatory approaches like AHP could also be valid, although they become challenging to implement with a large number of indicators.

Due to the different methods of index construction used, direct comparisons between the results of this study and others are not feasible. However, there are some general findings that can be discussed across Western European studies. Marzi et al. developed a disaster resilience index for Italy, which was inspired by the BRIC but differed significantly in its methodological approach [99]. This study used three different normalisation techniques and incorporated 128 sets of weights generated through Ordered Weighted Averaging (OWA); a measure of ‘OR-NESS’ was used to evaluate aggregation of the index. The complexity of this methodology is in sharp contrast to the BRIC approach and is likely to be less accessible to policymakers. One of the findings from this study was higher resilience in the more economically developed northern regions of Italy compared to the south of the country. In another methodologically complex paper, the BRIC was adapted for Germany by applying the Trapezoidal Fuzzy Decision-Making Trial and Evaluation Laboratory (DEMATEL) methodology to incorporate experts’ judgements in indicator weighting [100]. This study also found an association between higher resilience and urbanicity and reported that the least resilient counties were in the north and east of Germany. Finally, a much closer adaptation of the BRIC carried out for Norway reported that most of the municipalities with high resilience scores were in large urban areas [90]. This study explored the relationship between vulnerability and resilience, finding these to be negatively correlated with a similar magnitude to the relationship between the CRI and IMD in this paper.

### 4.2. Implications for Policy and Practise

The results of this study show that community resilience is spatially patterned in England, and therefore policymakers should prioritise targeted interventions to address this. The UK Government recently published a new Resilience Framework with the following three underpinning principles: a shared understanding of risk, increased emphasis on preparation and prevention, and empowering the ‘whole of society’ in contributing to resilience [2]. The policy includes a commitment to strengthening Local Resilience Forums (LRFs) and to developing a measurement of socioeconomic resilience by 2025. The CRI aligns with these goals and could be utilised or adapted to meet the government’s objective of creating a measurement tool. By incorporating the CRI, policymakers can better guide resilience-building initiatives and ensure that efforts are data-driven and effectively targeted.

The stated aim of the UK Government’s Levelling Up policy is to ensure that all communities have equitable access to physical, human, intangible, financial, social, and institutional capitals, thus reducing spatial disparity and enabling places to thrive [20]. The CRI closely mirrors these capitals and could play a role in supporting decisions on resource allocation, specifically in the context of levelling up funding. Concerns have been raised regarding the allocation of these funds, including that they are not being distributed equitably according to need [101]. By providing a detailed and multidimensional assessment of community resilience, the CRI could be used to identify priority areas, highlight specific areas of deficits, guide the equitable allocation of funding, and measure impact over time by longitudinal use of the CRI. A BRIC-based Community Resilience Index has been used elsewhere to model public health resource allocation [102].

In addition to national policy, the idea of using community resilience as a policy tool at the local government level has been explored. Platts-Fowler and Robinson use case studies and a review of the literature to investigate how community resilience could be operationalised at the local authority level [60]. They conclude that community resilience is a valuable policy tool with the potential to improve both resilience and well-being and highlighted the need for a comprehensive measurement framework and bottom–up approaches to support community engagement. There is further support for the idea of incorporating community resilience into local and regional policies from Ellis et al. [63]. They outline a model in which building community resilience leads to improved health outcomes and reduces structural inequalities.

The CRI provides the first detailed assessment of community resilience in England at a local authority level and could serve as a tool for local governments, LRFs, and other place-based organisations. By offering quantitative data, the CRI could guide the implementation of targeted interventions tailored to the specific needs of communities. Moreover, the CRI has the potential to be integrated with bottom–up data collected through participatory approaches. This integration would allow for a more nuanced understanding of local resilience dynamics, ensuring that interventions are not only data-driven but also reflective of the lived experiences and insights of local residents.

In this study, health indicators were intentionally excluded from the CRI because health is considered an outcome of community resilience rather than a cause. However, the relationship between community resilience and health may be more complex than this, with a degree of interdependence. The CRI captures multiple dimensions that align closely with the social determinants of health, which are the conditions in which people are born, grow, live, work, and age. These determinants include factors like employment, education, social and community context, and housing [103]. In measuring these components, the CRI is also assessing the environment that shapes health outcomes. Unlike the IMD, which includes health indicators within its framework, the CRI provides an independent measure that could be used to explore associations between community resilience and health outcomes. This distinction makes the CRI a potentially valuable tool for examining inequalities in access, experience, and outcomes of healthcare, offering a more profound and nuanced understanding of the factors that contribute to health inequalities.

## 5. Conclusions

The CRI scores revealed significant spatial patterns, with coastal, rural, and northern areas of England generally observed to have lower resilience. The CRI showed a moderate negative correlation with IMD, indicating that it adds value by providing new information rather than simply serving as an inverse measure of deprivation. The sensitivity analysis suggests that the CRI’s data-driven approach to index construction is robust and highlights the importance of empirical methodologies in developing composite indices. Despite its strengths, the CRI has limitations, including the use of administrative boundaries, which may not align with individual perceptions of community, and the challenge of capturing subjective aspects of resilience such as social capital through objective measures. Additionally, the data-driven approach reduces the scope for participatory input.

This study highlights the challenges of validating composite indices like the CRI. One area for future research is external validation of the index, including longitudinal studies to assess changes over time and compare these with actual community responses to various stresses and shocks. There is also the potential to incorporate participatory approaches in a future iteration of the CRI, for example, in relation to indicator selection and weighting decisions, and to interpret the data alongside bottom–up intelligence from local communities.

The CRI is a novel measure, and as such, there are unanswered questions regarding its validity and application. One avenue for future research would be to build on this study by incorporating temporal analyses to explore how community resilience changes over time. This would allow a dynamic understanding of resilience and its evolution in response to various stressors and interventions. Two potential applications of the CRI have been discussed, guiding decisions on resource allocation and exploring health outcomes. The CRI, by providing a detailed and multidimensional assessment of community resilience, could be used at a national level to identify priority areas for funding allocation and at a local level to highlight specific domains for targeted action. Future research utilising the CRI to study associations with health outcomes could enhance our understanding of how resilient communities foster better health and well-being, providing insights for public health interventions and policies. Thus, the CRI offers a framework for assessing community resilience across multiple dimensions, supporting targeted interventions and informed decision-making to enhance resilience and reduce spatial inequalities in England.

## Figures and Tables

**Table 1:** OECD framework for composite index construction.

Step	Description
1—Theoretical framework	A theoretical framework should be used as the basis for the selection and combination of variables into a meaningful composite indicator.
2—Data selection	Indicators should be selected based on the analytical soundness, measurability, country coverage, and relevance of the indicators to community resilience.
3—Imputation of missing data	Imputation methods should be used to estimate missing values if this is required to provide a complete dataset.
4—Multivariate analysis	The overall structure of the dataset should be assessed using an appropriate multivariate method to assess its suitability and guide subsequent methodological choices.
5—Normalisation	Normalisation should be carried out to make the variables comparable.
6—Weighting and aggregation	Indicators should be weighted and aggregated with respect to the theoretical framework and data properties.
7—Uncertainty and sensitivity bias	Uncertainty/sensitivity analyses should be undertaken to assess the robustness of the composite indicator in terms.
8—Back to the data	Identify if the composite indicator results are overly dominated by a few indicators and explain the relative importance of the sub-components of the composite indicator.
9—Links to other indicators	Correlate the composite indicator with existing (simple or composite) indicators.
10—Visualisation of results	Use an appropriate visualisation technique that communicates the most information clearly and accurately.

**Table 2:** Summary of themes from the analysis of community resilience indicators.

Dimension (Number of Themes)	Themes
Social (10)	Demographic, Health/well-being, Education/skills, Language/ Communication, Transport, Food, Civic engagement, Labour market, Community cohesion, and Other
Economic (8)	Labour market, Equity, Income/savings/debt, Commerce/retail, Housing, Demographic, Economic output, and Other
Institutional (8)	Local government, Mitigation, Disaster-specific, Civic engagement, Emergency services, Demographic, Energy/water, and Other
Infrastructure (9)	Housing, Transport, Health/well-being, Shelter capacity, Language/ Communication, Education/skills, Energy/water, Emergency services, and Other
Environmental (9)	Land type/use, Green space/forests, Disaster-specific, Waste/pollution, Soil/erosion, Energy/water, Food, Climate/elevation, and Other
Community capital (9)	Demographic, Civic engagement, Political engagement, Religion, Health/well-being, Education/skills, Culture/sports, Labour market, and Other

**Table 4:** Loading scores and eigenvalues from principal components analysis.

Sub-Index	Description	Variance Explained (%)	Variables (Mean Squared Loading)
1	Access and Infrastructure	12.54	Car availability (0.07)
			Population density (0.06)
			Household overcrowding (0.06)
			Age dependency ratio (0.06)
			Travel time to key services (0.05)
			Public health grant (0.05)
			Distance to sport and leisure facilities (0.05)
			Private outdoor space (0.04)
			Employment (0.04)
2	Economic Well-being and Opportunity	7.98	Housing affordability (0.09)
			Adult skills (0.08)
			Child poverty (0.08)
			Fuel poverty (0.07)
			Gross disposable household income (0.07)
			Electoral turnout (0.06)
			Digital propensity (0.05)
			Pupil absence (0.04)
			Gross value added (0.04)
			Inward migration (0.04)
			Economic inactivity (0.04)
3	Social Capital and Connectivity	3.53	SME loans (0.13)
			Broadband access (0.09)
			Lottery funding (0.08)
			Charities (0.05)
			Air pollution (0.04)
			Noise complaints (0.04)
			Public green space (0.04)
			Assets of community value (0.02)
			High street vibrancy (0.02)
4	Diversity and Inclusion	2.37	Religious affiliation (0.15)
			English language proficiency (0.12)
			Living alone (0.09)
			Municipal spending on culture (0.07)
			Employment sector diversity (0.06)
			Households in temporary accommodation (0.04)
			Life satisfaction (0.03)
5	Equity and Stability	1.60	Non-ringfenced reserves (0.18)
			Core spending power (0.17)
			IMD gap (0.15)
			School quality (0.06)
			Gender pay gap (0.05)
			Gini index (0.05)
			Population churn (0.04)
			Food insecurity (0.02)

**Table 5:** Geographical analysis of Community Resilience Sub-Index scores.

	Number of LADs	Composite Resilience Index	Sub-Index 1 Access and Infrastructure	Sub-Index 2 Economic Well-Being and Opportunity	Sub-Index 3 Social Capital and Connectivity	Sub-Index 4 Diversity and Inclusion	Sub-Index 5 Equity and Stability
**Regions**							
East Midlands	35	79.9 (8.9)	34.1 (5.1)	20.9 (6.3)	9.0 (1.5)	9.3 (1.7)	6.7 (1.7)
East of England	45	80.9 (11.4)	31.2 (4.0)	24.3 (7.3)	9.5 (1.3)	8.7 (1.5)	7.3 (1.2)
London	32	95.2 (6.2)	36.7 (1.8)	29.0 (4.8)	11.8 (2.3)	9.3 (1.5)	8.4 (0.7)
North East	12	77.5 (5.6)	35.4 (3.9)	16.1 (3.8)	8.7 (2.0)	10.1 (1.6)	7.2 (1.4)
North West	39	84.4 (9.1)	35.3 (5.1)	21.0 (6.4)	10.2 (1.6)	11.0 (1.7)	7.0 (1.1)
South East	64	87.3 (11.4)	31.4 (4.4)	29.1 (7.2)	10.0 (1.6)	9.3 (1.4)	7.5 (1.2)
South West	29	78.0 (9.7)	28.1 (6.2)	24.2 (5.2)	10.5 (2.1)	8.4 (1.4)	6.8 (1.4)
West Midlands	30	79.5 (7.2)	33.6 (4.5)	18.9 (6.6)	9.6 (1.7)	10.3 (1.5)	7.1 (1.2)
Yorkshire and The Humber	21	75.2 (8.0)	31.0 (5.7)	19.0 (7.3)	9.1 (1.7)	9.3 (1.9)	6.8 (1.0)
*p*-value (ANOVA)		<0.001	<0.001	<0.001	<0.001	<0.001	<0.001
**North–South**							
Midlands and South	235	83.9 (11.2)	32.3 (5.1)	25.0 (7.5)	10.0 (1.9)	9.2 (1.6)	7.3 (1.4)
North	72	80.6 (9.3)	34.1 (5.4)	19.6 (6.5)	9.6 (1.8)	10.3 (1.9)	7.0 (1.1)
*p*-value (*t*-test)		0.022	0.014	<0.001	0.16	<0.001	0.030
**Inland–Coastal**							
Inland	245	84.9 (10.5)	33.1 (5.0)	24.9 (7.7)	9.9 (1.9)	9.6 (1.6)	7.4 (1.3)
Coastal	62	76.0 (9.3)	31.5 (5.9)	19.3 (5.5)	9.8 (1.8)	8.8 (2.0)	6.7 (1.0)
*p*-value (*t*-test)		<0.001	0.032	<0.001	0.50	<0.001	<0.001
**Urban–Rural**							
Predominantly Urban	174	85.1 (10.5)	35.5 (3.2)	22.9 (7.9)	10.2 (2.1)	9.2 (1.7)	7.3 (1.4)
Urban with Significant Rural	50	83.2 (10.6)	31.3 (4.8)	25.6 (7.6)	9.3 (1.8)	9.7 (1.8)	7.3 (1.3)
Predominantly Rural	83	79.1 (10.7)	27.8 (4.7)	24.4 (6.7)	9.8 (1.4)	9.9 (1.6)	7.2 (1.2)
*p*-value (ANOVA)		<0.001	<0.001	0.053	0.012	0.005	0.69

**Table 6:** Correlation coefficients between Community Resilience Index and Index of Multiple Deprivation.

Variables	IMD
CRI: composite index	−0.564 *
Sub-Index 1: Access and Infrastructure	0.154 *
Sub-Index 2: Economic Well-being and Opportunity	−0.821 *
Sub-Index 3: Social Capital and Connectivity	0.030
Sub-Index 4: Diversity and Inclusion	−0.226 *
Sub-Index 5: Equity and Stability	−0.249 *

* shows significance at *p* < 0.05.

## Data Availability

The datasets used to generate the Community Resilience Index (CRI) for England are available in a GitHub repository, [https://github.com/Christine-L-Camacho/CRI_England]. This excludes the data for two indicators, which were used under licence/special agreement for the current study and so are not publicly available. The data for the retail vacancies indicator may be accessed under licence from the Consumer Data Research Centre, and the data on assets of community value can be obtained from Plunkett UK. All data generated from this study, namely the CRI and its sub-indices at local authority district level, are included in this published article and its Supplementary Information Files.

## Supplementary Materials

The following supporting information can be downloaded at: https://www.mdpi.com/article/10.3390/ijerph21081012/s1. Table S1. Summary of indicator themes and dimensions from systematic review of indicators (*n* = 1024); Table S2. Index and sub-index score for English local authority districts (*n* = 307); Table S3. Community Resilience Index and Index of Multiple Deprivation outliers; Table S4. Sensitivity analysis comparing weighted and unweighted CRI scores.
