# Supplementary material for: Adapting the Baseline Resilience Indicators for Communities (BRIC) Framework for England: Development of a Community Resilience Index
Source: Int J Environ Res Public Health. 2024 Aug 1;21(8):1012. doi: 10.3390/ijerph21081012 (PMC11354088; doi:10.3390/ijerph21081012)
Supplement: Supplementary file 1 [file ijerph-21-01012-s001.zip › ijerph-3105164-supplementary.pdf]

Supplementary Material

Table 1 - Summary of indicator themes and dimensions from systematic review of indicators (n=1024)

SYSTEMATIC REVIEW OF INDICATORS (n=1024)		
**Theme**	**Number**	**Summary of indicators**
**Social (n=256)**		
Demographic	66	Age, ethnicity, sex, immigration, population density, urbanicity
Health/wellbeing	46	Health insurance coverage, physical/mental disability, health facilities per capita, medical staff per capita,
Education/skills	36	Language competence, educational equity, qualifications, adult education programmes, literacy, teachers per capita
Language/ communication	32	Households with access to phone/radio/internet, English language proficiency
Transport	20	Access to vehicles, cars per capita
Food	9	Food security, access to fortified foods, food production/capita
Civic engagement	7	Number of civic organisations per capita, participation in voluntary work
Labour market	7	Public sector employment, unemployment, child labour, employment in professional/high skill jobs
Community cohesion	6	Community/social trust, social networks, community values, cooperation in disaster response
Other	27	Themes: Social services, Equity, Disaster-specific, Religion, Income/savings/debt, Culture/sports, Labour market, Mitigation, Political engagement, Crime
**Economic (n=215)**		
Labour market	63	Unemployment/employment, sector-specific employment, minimum wage rate, economic activity
Equity	28	Female labour force participation, gender pay gap, Gini coefficient, educational differences by ethnicity
Income/savings /debt	27	Annual income, average salary, household income, savings, loans/capita, receipt of welfare benefits,
Commerce/retail	27	Business size, commercial establishments per capita, lending institutions per capita, retail sale turnover
Housing	24	Owner occupied housing, property value, housing affordability, overcrowding
Demographic	9	Economic dependency ratio, population density, urbanicity, socio-economic disadvantage, working age population
Economic output	8	GDP per capita, GDP growth rate, industry specific contribution to GDP
Other	29	Themes: Health/wellbeing, Food, Local government, Productivity, Disaster-specific, Education/skills, Energy/water, Mitigation, Transport
**Institutional (n=137)**		
Local government	24	Political fragmentation, land use/zoning standards, proximity to seat of government, spending on public safety/municipal services, fiscal stress
Mitigation	21	Spend on mitigation projects per capita, flood insurance coverage, emergency/hazard mitigation plans, household/employment insurance coverage
Disaster-specific	15	Presidential disaster declarations, awareness of evacuation routes/shelters, earthquake education, flood warnings
Civic engagement	12	Citizen corps programme engagement, community participation, participation in hazard reduction programmes, volunteering
Emergency services	10	Spend on emergency services, number of ambulances/emergency services stations, first responder programmes
Demographic	9	Population distribution, population change
Energy/water	8	Access to electricity/drinking water, gas/electric cooking capability, proximity to nuclear power plant
Other	38	Themes: Housing, Education/skills, Food, Health/wellbeing, Employment, Transport, Language/communication, Equity, Water systems, Crime, Shelter capacity, Religion, Political engagement, Commerce/retail
**Infrastructure (193)**		

Housing	37	Time period houses built, type of housing, age/height of buildings, overcrowding, owner occupied housing, proximity to hazards, rental/mortgage costs
Transport	35	Highway density, length of rail/road network, road egress points/capita, public transport provision, vehicle registrations/capita, road traffic accidents per capita
Health/wellbeing	31	Hospital beds per capita, distance to health facilities, medical staff per capita, vacant hospital beds
Shelter capacity	21	Hotels/motels per capita, bed occupancy rate of hotels/motels, emergency shelter capacity, vacant housing units for rent
Language/ communication	17	Households with access to phone/radio/internet, mobile phones per capita
Education/skills	16	Schools/capita, schools/land area
Energy/water	14	Energy consumption per capita, water consumption per capita, gas/electric cooking capability, access to electricity/drinking water/sanitation, stormwater drainage
Emergency services	7	Fire/police/ambulance stations per capita, distance to fire/police station, firefighters/police officers per capita
Other	15	Themes: Demographics, Disaster-specific, Social services, Culture/sports, Crime, Waste/pollution, Commerce/retail, Employment, Labour market, Green space/forests
**Environmental (106)**		
Land type/use	24	Percentage of land in wetlands, urban areas, developed open spaces, protected status, change in land use type
Green space/forests	13	Parks/urban green areas, forest coverage, parks per capita
Disaster-specific	11	Earthquake frequency, flooding, loss causing hazards, environmental emergencies, flood/landslide zones, economic loss from disasters
Waste/pollution	10	Sewage treatment, waste disposal, recycling ratio, industrial waste management
Soil/erosion	10	Perviousness of soil, erosion rates
Energy/water	10	Energy consumption/capita, water consumption per capita, water supply stress index
Food	10	Agricultural holdings per capita, farms marketing products through community supported agriculture, grain output per capita, arable land
Climate/elevation	10	Mean elevation, number of rainy days/rainstorm frequency, high temperature, flood duration, loss causing extreme weather events
Other	8	Themes: Water systems, transport, housing
**Community capital (117)**		
Demographic	26	Migration (domestic and international), population born in place, population working outside of country
Civic engagement	19	Civic organisations per capita, social advocacy organisations per capita, volunteers per capita, red cross volunteers per capita
Political engagement	15	Electoral turnout
Religion	13	Religious affiliation per capita, religious organisations per capita
Health/wellbeing	9	Absence of psychopathologies, quality of life/life satisfaction, suicide per capita, counselling services, physical/mental disability
Education/skills	8	Childcare facilities per capita, universities/capita, Red Cross training participation per capita, local understanding of risk
Culture/sports	7	Sports facilities per capita, cultural/entertainment facilities per capita
Labour market	6	Employment, sector-specific employment
Other	14	Themes: Crime, community cohesion, local government, commerce/retail, language/communication, equity

Table 2 – Index and sub-index score for English Local Authority Districts (n=307)

Local authority name	Sub index 1	Sub index 2	Sub index 3	Sub index 4	Sub index 5	CRI
Tendring	21.2	11.6	7.6	8.2	4.8	53.3
Fenland	23.8	12.5	7.1	6.2	6.3	55.8
East Lindsey	20.3	10.8	8.8	11.0	5.7	56.6
Scarborough	17.1	14.1	10.7	9.9	6.0	57.7
Great Yarmouth	25.6	10.8	11.1	6.2	6.6	60.3
King's Lynn and West Norfolk	22.5	14.0	8.6	9.1	6.6	60.8

Boston	25.0	12.0	9.1	8.8	7.3	62.2
Thanet	28.2	11.7	9.0	6.7	7.1	62.8
North Devon	16.8	19.5	11.4	8.3	7.2	63.2
Torridge	19.2	16.5	12.4	8.7	6.5	63.3
Folkestone and Hythe	22.2	18.3	7.5	9.2	6.9	64.1
North Norfolk	22.8	15.5	9.0	10.0	7.7	65.0
North East Lincolnshire	36.3	11.3	6.9	6.8	5.4	66.7
Dover	25.3	18.7	7.8	7.8	7.2	66.7
Isle of Wight	23.2	16.6	11.1	8.5	7.5	66.8
Teignbridge	22.9	22.1	5.9	10.2	5.8	66.9
Breckland	26.3	17.0	9.5	8.1	6.0	67.0
Torbay	29.3	14.8	9.5	7.3	6.2	67.1
Doncaster	35.7	10.4	7.1	8.4	5.6	67.1
Rother	22.4	19.7	9.6	9.9	5.6	67.2
Kingston upon Hull	37.4	10.1	7.6	5.6	6.8	67.5
Wyre Forest	30.6	12.2	9.2	9.0	6.8	67.9
South Holland	28.6	15.0	9.0	9.3	6.1	68.0
Swale	27.0	17.8	9.9	8.2	5.2	68.0
Bradford	30.5	11.3	9.7	9.1	7.7	68.2
East Suffolk	25.4	18.1	10.2	7.7	6.7	68.3
Lincoln	31.8	15.9	9.4	6.1	5.1	68.4
West Devon	21.5	19.6	10.1	10.0	7.3	68.5
Cornwall	22.1	18.1	12.0	8.1	8.7	69.0
Stoke-on-Trent	36.7	9.5	8.6	8.2	6.3	69.3
Hastings	29.5	14.3	12.1	6.6	6.8	69.4
Forest of Dean	24.6	21.1	10.4	8.2	5.3	69.5
Sedgemoor	25.6	20.1	10.7	7.9	5.3	69.6
Pendle	33.8	13.8	7.7	9.5	4.8	69.6
Redcar and Cleveland	32.8	12.7	6.9	11.2	6.0	69.6
Herefordshire	21.9	18.9	12.1	8.1	8.7	69.7
Somerset West and Taunton	21.0	21.3	12.6	9.3	5.5	69.7
East Staffordshire	31.3	16.5	6.3	9.0	6.8	69.9
Middlesbrough	35.9	12.0	8.1	8.4	5.6	70.0
North Lincolnshire	33.8	14.7	5.7	10.5	6.0	70.7
Allerdale	26.2	18.4	10.6	10.6	5.0	70.8
Havant	28.2	20.4	9.7	7.8	4.9	71.0
Arun	27.3	20.0	9.5	7.0	7.2	71.0
Shropshire	23.2	19.1	10.8	10.7	7.6	71.5
Carlisle	26.8	18.0	12.3	9.5	5.0	71.7
Norwich	31.3	16.6	11.8	5.9	6.3	71.8
Mid Devon	22.2	22.9	10.8	7.9	8.1	71.9
Rotherham	37.9	11.2	8.3	8.7	5.7	71.9
Sheffield	30.8	15.2	10.3	7.5	8.5	72.3
West Lindsey	26.9	19.4	8.7	11.1	6.4	72.5
Walsall	32.7	9.2	10.6	11.4	8.6	72.5
Calderdale	30.6	19.0	9.8	7.9	5.2	72.5
Peterborough	31.5	17.5	9.7	8.2	5.9	72.8
Ipswich	33.5	15.0	11.4	5.8	7.2	73.0
Kirklees	31.8	16.6	8.4	9.0	7.5	73.3
Telford and Wrekin	32.9	16.0	8.8	8.7	6.9	73.3
Copeland	26.6	19.7	9.3	11.1	6.7	73.3
Bassetlaw	33.6	13.1	9.0	10.0	7.8	73.5
Ryedale	23.1	22.3	10.6	10.5	7.1	73.5

Birmingham	33.0	11.8	9.3	10.0	9.5	73.6
Blackpool	35.8	11.2	11.9	9.0	5.7	73.6
Rochdale	34.3	12.3	9.3	9.3	8.3	73.6
Mendip	25.2	24.1	10.4	7.9	6.5	74.0
Ashfield	40.7	13.2	7.4	6.9	6.3	74.5
Gosport	29.9	20.5	10.4	8.9	4.9	74.6
Newcastle-under-Lyme	35.4	16.1	7.2	11.0	5.0	74.8
Southend-on-Sea	34.9	17.1	10.2	6.2	6.6	75.0
Dorset	23.1	23.6	11.0	9.6	7.8	75.1
Northumberland	25.3	19.0	10.6	11.2	9.0	75.2
Derby	35.1	15.7	10.0	8.5	5.9	75.2
Hartlepool	38.1	12.4	7.2	11.2	6.5	75.3
Wakefield	37.8	15.3	6.0	8.7	7.6	75.4
Mansfield	38.3	13.0	11.1	7.9	5.1	75.4
Braintree	29.5	23.2	7.0	8.2	7.6	75.5
Plymouth	35.8	16.9	8.1	8.1	6.8	75.6
East Riding of Yorkshire	25.5	21.6	9.6	11.9	7.0	75.6
Chesterfield	38.0	14.7	10.5	9.6	2.9	75.6
West Suffolk	28.9	22.6	9.6	6.8	7.7	75.7
Luton	34.6	14.5	9.9	9.8	7.0	75.8
Canterbury	26.1	22.5	9.5	9.4	8.3	75.8
Oldham	34.2	12.1	9.9	11.6	8.1	75.9
Newark and Sherwood	33.4	20.4	6.7	9.3	6.2	76.0
Thurrock	32.2	20.5	6.2	9.5	7.7	76.1
Barnsley	38.9	12.4	8.4	8.1	8.3	76.2
Nottingham	34.2	15.4	11.2	6.9	8.5	76.2
Burnley	36.2	12.4	10.4	10.7	6.9	76.5
Babergh	28.9	23.1	9.0	9.4	6.2	76.5
Nuneaton and Bedworth	39.0	12.9	8.8	10.0	5.8	76.5
Maldon	30.0	22.6	8.9	9.1	6.1	76.7
Darlington	36.2	17.9	7.0	10.0	5.9	77.0
Gloucester	35.7	20.5	10.1	7.7	3.0	77.0
Ashford	27.0	22.0	10.6	10.1	7.3	77.0
Melton	29.0	23.7	8.0	9.9	6.4	77.1
Wolverhampton	34.4	12.1	11.7	11.0	7.9	77.1
Lewes	25.3	26.5	11.4	7.7	6.4	77.3
Dudley	39.3	10.9	10.0	10.9	6.1	77.3
Coventry	34.3	17.6	10.2	8.6	6.7	77.4
Sandwell	36.2	10.7	10.8	10.6	9.2	77.5
Eastbourne	28.1	22.0	14.0	7.2	6.2	77.5
North East Derbyshire	36.1	18.7	7.4	9.4	5.8	77.5
Staffordshire Moorlands	31.5	17.9	8.8	12.2	7.1	77.5
Stockton-on-Tees	34.6	20.3	5.6	11.3	5.9	77.6
Hyndburn	37.7	13.9	9.5	10.8	5.8	77.7
Sunderland	37.3	13.2	8.4	9.6	9.4	77.8
Chichester	22.8	27.4	10.0	9.2	8.5	77.9
North Somerset	32.4	26.6	7.2	6.2	5.6	78.0
Gateshead	35.7	14.5	10.6	9.4	7.8	78.0
North Northamptonshire	36.1	19.1	6.6	7.0	9.6	78.3
South Hams	21.2	27.9	11.8	9.7	7.8	78.4
Leeds	32.8	19.9	9.6	8.9	7.2	78.4
Leicester	33.6	14.0	11.2	10.7	9.2	78.6
Swindon	35.4	26.6	4.8	6.3	5.7	78.9

East Devon	25.1	26.2	10.0	11.3	6.3	78.9
County Durham	35.0	14.6	8.0	12.3	9.1	79.0
Newcastle upon Tyne	34.0	18.6	12.5	6.8	7.1	79.0
Colchester	30.0	25.6	7.8	9.0	6.5	79.0
Tamworth	38.1	17.2	7.7	9.3	6.7	79.0
Castle Point	38.0	17.0	9.1	8.7	6.3	79.2
Eden	24.8	23.7	12.7	10.7	7.3	79.2
Adur	32.2	22.4	11.1	6.4	7.3	79.3
Southampton	36.2	21.0	9.5	6.2	6.4	79.3
Barking and Dagenham	35.2	15.8	9.5	10.6	8.2	79.3
Bolsover	40.2	14.6	9.1	7.5	8.0	79.4
South Tyneside	37.9	13.5	10.1	11.0	7.0	79.4
South Somerset	27.6	24.2	10.1	10.6	7.2	79.6
Preston	32.2	20.1	10.5	10.9	6.0	79.6
Exeter	32.3	22.4	12.5	7.2	5.2	79.7
Tameside	39.1	15.2	9.6	8.3	7.6	79.7
Rossendale	35.5	20.5	7.2	10.1	6.7	79.9
North Warwickshire	33.8	18.3	10.7	12.3	5.0	80.1
Blackburn with Darwen	38.5	13.7	10.8	11.5	5.7	80.2
Epping Forest	28.2	27.8	8.8	10.1	5.3	80.4
Amber Valley	37.6	20.1	8.6	9.4	4.7	80.4
West Northamptonshire	29.5	25.7	8.1	7.7	9.5	80.5
Basildon	32.9	20.6	10.9	7.7	8.4	80.5
Bolton	35.8	15.7	11.4	9.3	8.4	80.6
Redditch	37.7	17.7	9.5	9.4	6.3	80.6
North West Leicestershire	35.6	23.1	7.9	8.5	5.5	80.7
New Forest	26.9	27.2	9.6	9.4	7.8	80.9
Erewash	42.3	17.9	9.7	7.8	3.1	80.9
Knowsley	35.0	15.3	11.0	12.4	7.2	80.9
Harlow	34.7	18.5	11.3	7.4	9.1	81.0
Bournemouth, Christchurch and Poole	32.2	22.8	11.5	7.5	7.3	81.2
Richmondshire	26.2	27.8	9.9	12.0	5.4	81.3
Malvern Hills	27.3	25.2	9.9	11.4	7.7	81.4
Liverpool	34.5	16.9	11.8	10.4	7.9	81.4
South Staffordshire	33.2	22.8	6.5	13.4	5.6	81.5
High Peak	34.6	23.7	9.4	8.7	5.1	81.6
Barrow-in-Furness	36.7	21.3	8.0	10.7	5.0	81.6
Salford	35.1	19.8	10.8	7.8	8.0	81.6
Stevenage	35.9	23.6	9.4	7.3	5.4	81.7
Portsmouth	37.4	20.8	8.6	7.4	7.5	81.7
Craven	26.4	28.1	11.0	9.9	6.6	82.0
Medway	37.9	20.8	7.6	8.7	7.1	82.1
South Kesteven	32.3	24.0	9.1	10.0	6.9	82.3
Gravesham	33.7	20.9	11.5	9.4	6.9	82.3
South Lakeland	23.3	29.8	10.9	10.1	8.4	82.6
Charnwood	32.7	24.2	9.9	8.6	7.2	82.6
Mid Suffolk	30.5	25.8	9.9	9.6	7.2	83.0
Rochford	34.7	24.4	7.6	9.2	7.2	83.1
East Cambridgeshire	30.5	30.1	9.1	7.6	5.9	83.2
York	32.7	27.1	9.2	7.2	7.3	83.4
Stafford	31.5	24.2	8.0	12.2	7.5	83.5
Derbyshire Dales	28.4	26.0	9.3	12.3	7.6	83.6
Maidstone	30.2	27.4	9.4	9.3	7.3	83.6

Crawley	34.1	23.8	8.4	11.2	6.4	83.8
Hinckley and Bosworth	36.1	24.2	7.8	8.2	7.6	83.9
Welwyn Hatfield	28.0	29.6	9.0	9.8	7.5	83.9
Cannock Chase	41.7	14.1	9.3	11.5	7.5	84.0
Lancaster	30.5	23.5	12.0	10.5	7.8	84.2
Broxbourne	33.1	24.2	10.5	9.7	6.8	84.3
Milton Keynes	32.0	29.6	7.0	8.3	7.4	84.3
Cambridge	30.2	28.6	10.8	7.2	7.6	84.4
Sefton	35.9	19.1	10.8	11.4	7.2	84.5
Manchester	34.9	19.5	11.7	9.2	9.2	84.5
Halton	39.8	20.2	6.5	11.1	7.0	84.6
Wiltshire	26.7	29.5	10.7	10.9	6.8	84.6
Bedford	32.9	25.7	11.1	9.2	5.9	84.6
Rugby	33.1	27.8	7.1	8.9	7.8	84.7
Worthing	32.7	28.6	11.2	6.7	5.6	84.7
St. Helens	41.4	16.2	9.3	11.6	6.4	84.9
Wychavon	31.6	23.5	11.0	10.7	8.1	85.0
North Kesteven	34.5	25.2	6.8	12.1	7.0	85.5
Solihull	34.0	25.7	8.8	10.8	6.4	85.7
Havering	34.0	24.7	8.9	10.1	8.1	85.7
Newham	36.6	20.0	10.4	11.2	7.5	85.8
Hambleton	25.7	28.7	12.2	13.0	6.5	86.1
East Hampshire	27.2	34.0	8.0	9.8	7.2	86.1
Gedling	40.1	23.8	7.2	8.9	6.5	86.4
Oxford	33.2	26.1	11.0	9.0	7.2	86.5
Oadby and Wigston	37.7	22.2	10.9	10.3	5.7	86.7
Wyre	33.7	22.0	9.6	14.4	7.2	86.8
Bath and North East Somerset	32.1	28.7	11.7	7.0	7.5	86.9
West Lancashire	33.1	24.7	10.2	13.4	5.9	87.1
Broxtowe	39.2	26.1	9.0	7.7	5.2	87.3
Wealden	30.6	28.0	10.8	9.8	8.2	87.4
Stratford-on-Avon	29.1	31.8	9.3	9.4	7.8	87.5
Rutland	25.7	30.6	10.5	13.8	6.9	87.6
Lichfield	33.3	25.6	10.6	11.2	7.3	87.9
Huntingdonshire	32.9	31.0	9.6	6.9	7.5	87.9
Brighton and Hove	35.6	28.8	12.1	5.7	5.8	88.0
Worcester	41.1	19.9	13.5	8.9	4.8	88.2
South Norfolk	33.0	27.2	8.5	10.7	8.8	88.2
Cotswold	25.6	33.3	11.2	9.9	8.2	88.2
Slough	34.9	24.2	11.6	9.5	8.2	88.4
Test Valley	29.7	34.4	7.7	10.6	6.0	88.4
Basingstoke and Deane	31.8	35.2	6.0	8.8	6.9	88.6
Wirral	39.2	20.5	11.0	12.0	6.1	88.8
Tunbridge Wells	27.4	33.0	11.5	9.9	7.0	88.8
Broadland	34.9	26.6	9.7	8.8	8.9	88.9
Wigan	42.3	17.4	9.0	12.1	8.0	88.9
Lewisham	36.4	27.1	8.9	8.0	8.4	88.9
Greenwich	36.1	27.2	7.5	9.2	9.0	88.9
Selby	33.6	30.3	9.1	9.4	6.7	89.1
Harrogate	26.7	30.8	10.9	12.3	8.4	89.1
Bexley	36.1	26.2	8.2	11.1	7.7	89.3
Mole Valley	26.1	37.2	12.0	9.0	5.1	89.4
Fylde	32.7	28.8	8.2	13.0	6.8	89.5

Bristol	37.6	23.7	14.2	5.9	8.2	89.6
Cheshire West and Chester	32.5	29.0	10.0	10.0	8.1	89.6
Horsham	29.1	35.1	9.0	9.0	7.6	89.9
Enfield	33.9	24.6	12.6	9.9	8.9	89.9
Dacorum	35.8	29.0	9.8	7.3	8.0	89.9
Winchester	24.2	36.4	10.7	11.1	8.0	90.5
Tandridge	28.1	32.8	10.1	11.4	8.5	90.8
Brent	35.3	24.1	12.3	9.1	10.2	91.1
Chelmsford	32.6	30.8	8.5	10.1	9.1	91.1
Sevenoaks	31.3	31.2	10.5	10.3	8.0	91.2
Cherwell	32.7	31.7	9.0	10.4	7.6	91.3
Hillingdon	36.6	26.9	9.0	11.2	7.7	91.3
Tewkesbury	32.5	32.8	9.8	9.2	7.0	91.4
Dartford	35.5	29.8	8.8	10.0	7.4	91.5
Uttlesford	30.9	31.2	8.8	11.1	9.6	91.5
Rushmoor	37.0	30.2	7.5	10.6	6.3	91.5
North Hertfordshire	32.2	34.4	8.5	8.2	8.3	91.6
Central Bedfordshire	38.3	30.0	7.4	8.1	7.9	91.7
Redbridge	34.3	26.9	11.6	10.6	8.5	91.9
North Tyneside	41.8	24.0	9.7	9.0	7.4	92.0
Warwick	31.4	32.5	11.1	7.9	9.0	92.0
Stroud	32.3	31.0	10.8	8.4	9.9	92.4
Waltham Forest	37.2	24.7	11.0	10.0	9.5	92.4
East Hertfordshire	31.4	34.0	9.5	9.3	8.4	92.6
Bury	39.4	20.9	14.1	10.6	7.8	92.8
Haringey	37.1	25.6	14.2	8.0	8.0	92.9
West Oxfordshire	30.6	33.6	11.0	10.1	7.8	93.0
Hertsmere	32.3	31.1	10.6	10.6	8.6	93.3
Croydon	35.5	28.5	11.4	10.3	7.8	93.3
Runnymede	32.0	34.0	9.2	10.3	8.1	93.6
Guildford	28.9	36.6	10.3	10.5	7.3	93.6
Harborough	30.3	32.3	12.0	11.1	7.9	93.6
Waverley	29.6	34.5	10.9	10.5	8.2	93.7
Cheshire East	32.5	32.4	11.0	10.6	7.3	93.8
Hackney	37.5	25.8	15.9	7.4	7.4	93.9
Tonbridge and Malling	36.1	30.7	8.8	10.2	8.3	94.1
South Derbyshire	41.6	28.7	6.2	10.1	7.5	94.1
Buckinghamshire	31.3	33.6	11.1	9.7	8.4	94.2
Reading	35.7	30.5	13.1	8.5	6.6	94.3
Stockport	39.4	27.3	11.7	9.4	6.6	94.3
Brentwood	31.9	33.4	10.6	11.2	7.4	94.5
Blaby	38.6	28.0	10.1	8.8	9.6	95.0
Hounslow	34.9	28.8	11.6	11.1	9.3	95.6
Harrow	35.2	29.9	12.3	9.6	8.8	95.8
Vale of White Horse	31.6	37.6	8.6	9.5	8.4	95.8
Camden	36.9	27.7	15.7	7.7	7.8	95.8
West Berkshire	30.1	36.5	11.5	8.4	9.4	95.9
Watford	34.9	30.5	12.3	10.3	7.9	96.0
Ealing	35.3	29.8	13.3	9.6	8.2	96.2
Mid Sussex	33.7	36.1	9.3	9.3	7.9	96.2
Tower Hamlets	37.4	29.6	12.4	9.0	7.9	96.2
Sutton	39.1	30.5	8.4	10.0	8.5	96.5
Spelthorne	34.3	32.8	10.8	10.1	8.6	96.6

Eastleigh	36.2	34.5	8.6	8.8	8.7	96.8
South Gloucestershire	38.7	33.4	8.3	7.6	8.8	96.8
Bromsgrove	38.2	29.3	11.1	12.0	6.5	97.2
Bromley	34.6	33.8	9.2	10.4	9.2	97.2
Windsor and Maidenhead	32.8	36.9	11.1	9.7	6.9	97.3
Islington	38.0	31.3	14.9	4.4	8.6	97.3
Cheltenham	37.8	31.5	14.3	7.4	6.7	97.6
Barnet	34.0	32.6	14.1	9.1	8.2	98.0
Southwark	38.2	30.5	13.2	8.1	8.0	98.0
Warrington	41.7	29.0	9.6	10.1	7.8	98.1
South Cambridgeshire	33.2	37.2	9.6	8.8	9.4	98.2
Chorley	42.1	26.1	8.9	14.5	6.8	98.3
Fareham	36.9	32.1	9.9	11.4	8.1	98.4
Bracknell Forest	39.3	36.5	7.5	8.2	8.0	99.5
Three Rivers	34.5	36.6	9.7	11.7	7.3	99.8
St Albans	33.3	36.7	10.2	11.3	8.4	99.9
Lambeth	39.6	31.6	12.8	7.2	8.7	99.9
Rushcliffe	35.1	36.8	7.9	10.4	10.0	100.2
South Oxfordshire	34.1	37.2	10.9	9.6	9.0	100.8
Hammersmith and Fulham	38.6	33.5	13.2	7.5	8.5	101.3
Kensington and Chelsea	37.5	32.8	14.1	9.8	7.4	101.5
Woking	35.5	36.5	9.0	11.3	9.2	101.6
Westminster	37.1	30.8	14.7	9.4	9.7	101.7
Merton	38.3	34.1	11.1	9.7	8.6	101.8
Trafford	41.7	31.6	11.7	10.2	7.1	102.2
Epsom and Ewell	35.5	35.4	11.6	11.5	8.6	102.6
Reigate and Banstead	32.8	36.3	13.1	10.7	9.9	102.8
Ribble Valley	35.5	35.3	10.8	14.8	7.6	104.0
Hart	35.8	39.4	9.1	9.9	10.7	105.0
Wandsworth	40.8	36.8	11.0	8.2	8.4	105.3
South Ribble	45.7	31.4	6.9	14.5	7.2	105.7
Kingston upon Thames	39.1	36.4	11.8	10.0	8.5	105.7
Richmond upon Thames	37.9	38.2	12.4	9.2	8.9	106.6
Surrey Heath	38.4	37.0	10.8	11.1	9.6	106.8
Wokingham	39.9	39.0	10.1	9.7	9.5	108.2
Elmbridge	36.0	38.6	12.6	11.8	9.8	108.9

The data in Table 2 is also available as a csv file with LAD codes in a GitHub repository.

Table 3 - Community resilience index and Index of Multiple Deprivation outliers

Local Authority	Region	IMD rank*	Predicted CRI score	Actual CRI score	Difference
**Positive Outliers – CRI higher than predicted from IMD**					
Lambeth	London	228	78.0	99.9	21.9
Hammersmith and Fulham	London	197	80.2	101.3	21.2
Islington	London	253	76.3	97.3	21.0
Kensington and Chelsea	London	184	81.1	101.5	20.5
Wandsworth	London	128	84.9	105.3	20.4
Southwark	London	232	77.8	98.0	20.2
Tower Hamlets	London	254	76.2	96.2	20.0
Hackney	London	285	74.1	93.9	19.8
Westminster	London	172	81.9	101.7	19.8
South Ribble	North West	106	86.4	105.7	19.3

**Negative Outliers - CRI lower than predicted from IMD**					
Fenland	East of England	229	78.0	55.8	-22.2
Tendring	East of England	271	75.1	53.3	-21.8
Scarborough	Yorkshire and The Humber	239	77.3	57.7	-19.5
Teignbridge	South West	116	85.7	66.9	-18.8
East Lindsey	East Midlands	267	75.3	56.6	-18.8
North Devon	South West	175	81.7	63.2	-18.5
King's Lynn and West Norfolk	East of England	214	79.0	60.8	-18.2
Boston	East Midlands	208	79.4	62.2	-17.2
North Norfolk	East of England	182	81.2	65.0	-16.2
Torridge	South West	210	79.3	63.3	-15.9
*High IMD rank indicates higher relative deprivation					

Table 4 - Sensitivity analysis comparing weighted and unweighted CRI scores

	CRI (rank)	CRI_{BRIC} (rank)	Difference
**Regions**			
East Midlands	179.4 (73.1)	173.5 (83.2)	5.9 (64.4)
East of England	166.3 (88.4)	185.2 (80.6)	-18.9 (42.8)
London	54.7 (41.0)	48.6 (29.9)	6.1 (25.2)
North East	207.3 (52.2)	153.9 (78.8)	53.4 (47.5)
North West	143.5 (76.5)	111.9 (73.5)	31.6 (55.1)
South East	120.2 (91.0)	135.9 (81.4)	-15.8 (48.3)
South West	197.4 (84.0)	216.0 (87.0)	-18.6 (35.9)
West Midlands	186.6 (67.1)	179.5 (72.3)	7.1 (63.1)
Yorkshire and The Humber	222.2 (67.1)	226.4 (56.8)	-4.2 (54.3)
p-value (ANOVA)	<0.001	<0.001	<0.001
**North-South**			
Midlands and South	146.9 (90.6)	154.5 (90.0)	-7.6 (49.5)
North	177.1 (78.7)	152.3 (85.3)	24.8 (56.7)
p-value (t-test)	0.011	0.85	<0.001
**Inland-Coastal**			
Inland	139.8 (86.5)	145.3 (88.5)	-5.5 (52.9)
Coastal	210.2 (74.6)	188.4 (81.8)	21.8 (47.7)
p-value (t-test)	<0.001	<0.001	<0.001
**Urban-Rural**			
Predominantly Urban	140.8 (87.9)	121.7 (80.0)	19.1 (49.8)
Urban with Significant Rural	148.1 (85.6)	165.3 (84.1)	-17.2 (50.3)
Predominantly Rural	185.3 (85.7)	214.9 (75.1)	-29.6 (43.6)
p-value (ANOVA)	<0.001	<0.001	<0.001
Higher rank = lower community resilience score			
